# A Multiple siRNA-Based Anti-HIV/SHIV Microbicide Shows Protection in Both *In Vitro* and *In Vivo* Models

**DOI:** 10.1371/journal.pone.0135288

**Published:** 2015-09-25

**Authors:** Sandhya Boyapalle, Weidong Xu, Payal Raulji, Subhra Mohapatra, Shyam S Mohapatra

**Affiliations:** 1 Department of Internal Medicine -Division of Translational Medicine and Nanomedicine Research Center, University of South Florida, Tampa, Florida, United States of America; 2 Department of Molecular Medicine, Morsani College of Medicine, University of South Florida, Tampa, Florida, United States of America; 3 James A Haley VA Hospital, Tampa, Florida, United States of America; 4 Transgenex Nanobiotech Inc, Tampa, Florida, United States of America; Central Michigan University School of Medicine, UNITED STATES

## Abstract

Human immunodeficiency virus (HIV) types 1 and 2 (HIV-1 and HIV-2) are the etiologic agents of AIDS. Most HIV-1 infected individuals worldwide are women, who acquire HIV infections during sexual contact. Blocking HIV mucosal transmission and local spread in the female lower genital tract is important in preventing infection and ultimately eliminating the pandemic. Microbicides work by destroying the microbes or preventing them from establishing an infection. Thus, a number of different types of microbicides are under investigation, however, the lack of their solubility and bioavailability, and toxicity has been major hurdles. Herein, we report the development of multifunctional chitosan-lipid nanocomplexes that can effectively deliver plasmids encoding siRNA(s) as microbicides without adverse effects and provide significant protection against HIV in both *in vitro* and *in vivo* models. Chitosan or chitosan-lipid (chlipid) was complexed with a cocktail of plasmids encoding HIV-1-specific siRNAs (psiRNAs) and evaluated for their efficacy in HEK-293 cells, PBMCs derived from nonhuman primates, 3-dimensional human vaginal ectocervical tissue (3D-VEC) model and also in non-human primate model. Moreover, prophylactic administration of the chlipid to deliver a psiRNA cocktail intravaginally with a cream formulation in a non-human primate model showed substantial reduction of SHIV (simian/human immunodeficiency virus SF162) viral titers. Taken together, these studies demonstrate the potential of chlipid-siRNA nanocomplexes as a potential genetic microbicide against HIV infections.

## Introduction

According to the most recent UNAIDS estimates, more than 78 million people have been infected with human immunodeficiency virus (HIV) and 39 million have died since the start of the AIDS epidemic. At the end of 2012, there were 35.2 million people living with HIV globally and there were 2.3 million new HIV infections in 2012 [1][2]. HIV type-1 (HIV-1) continues to spread mainly by heterosexual sex [3–5]. Although AIDS vaccine research has made great progress in the past 30 years and many neutralizing antibodies, CTL epitopes, and vaccine vectors have been discovered [6–13], there is no FDA-approved vaccine available currently. Hence, alternative prevention methods are needed to supplement educational and behavioral-modification programs.

Most HIV-1 infected individuals worldwide are women, and most acquire HIV infection during sexual contact. At present, the best strategy to accomplish blocking HIV mucosal transmission and local spread in the female genital tract involves design and implementation of topical microbicides that are used before sex and interfere with viral transmission. The microbicides prevent HIV-1 sexual transmission by either destroying the microbes or preventing them from infecting target cells. Some small molecules such as cellulose acetate 1,2-benzene-dicarboxylate (CAP), BMS-378806, which binds to the viral gp120 glycoprotein and prevents its attachment to the CD4 and CCR5 receptors, and CMPD167, a small molecule that binds to CCR5 to inhibit gp120 association, have been protective in non-human primates. Many natural products, such as genistein and curcumin were also found to inhibit HIV infection. Some algal lectins such as cyanovirin-N, scytovirin, and griffithsin exhibit high anti-HIV activity. Cyanovirin-N has been the most extensively investigated microbicide thus far. However, the lack of solubility and bioavailability has limited their clinical application and has led to failed clinical trials.

Chitosan, a natural biocompatible cationic polysaccharide derived from crustacean shells, has been used not only as a dietary supplement for weight loss, but has also shown antimicrobial properties [14, 15]. Importantly, chitosan has also shown anti-HIV properties that are similar to the action of microbicides, which kill or inactivate HIV, stop the virus entering human cells, inhibit HIV replication, and potentially enhance the body’s normal defense mechanisms against HIV [16, 17]. Thus, low molecular weight sulphated chitosan was shown to block viral entry and virus-cell fusion probably via disrupting the binding of HIV-1 gp120 to CD4 cell surface receptor [17]. Intravaginal mucoadhesive chitosan microspheres of tenofovir disoproxil fumarate (TDF) were also shown to be stable for months [18]. Also, thiolated chitosan was shown to enhance and retain topical microbicides longer, such as tenofovir [19]. Recently, chitosan oligomers conjugated with tripeptides (tryptophan, methionine and glutamine) were shown to actively inhibit the syncytia formation upon co-culture of HIV-infected and -uninfected C8166 cells [20].

We have investigated the use of chitosan as a tool in gene-silencing, using vector-driven expression of siRNAs, which have shown promise in treating viral infections [21–24]. The advantages of vector driven siRNA include ease of construction of plasmid vectors devoid of viral genes, stability and heat resistance, the possibility of combining siRNAs from virus and/or host genes to form a therapy cocktail, and ease of preparation and cost-effectiveness. The plasmid vectors used do not replicate in mammalian hosts and do not integrate into host genomes, yet they can persist in host cells and express the siRNAs for a period of days to weeks. Several reports indicated that by targeting viral proteins, cellular receptors or co-receptors, siRNAs can significantly inhibit HIV-1 replication in vitro. However, the use of such a siRNA approach for mucosal (vaginal) gene transfer in form of a microbicide has not been explored.

A major challenge of the early vaginal or rectal microbicides has been that they caused denudation of the mucosal epithelium promoting HIV and also potentially other viral and bacterial infections [25]. Because chitosan has anti-microbial and anti-HIV activities, wound-healing properties, no cytotoxic activity, and facilitates mucosal gene delivery, we used a combination of chitosan-based nanoparticles and siRNAs to prepare a potent microbicide in the present study. Herein we report a novel chitosan-lipid (chlipid) nanoparticles (CNs) to deliver plasmids encoding siRNAs for protection against HIV-1 infection *in vivo*. Based on our success using chitosan-nanoparticles to deliver siRNAs to mice, we tested a cocktail of anti-HIV siRNA nanoparticles for preventing SHIV-1 transmission in a macaque model in a pilot study. The results showed that the anti-HIV siRNA microbicide cocktail can be selectively delivered by CNs to HIV-susceptible cells in mucosal tissues.

## Materials and Methods

### Preparation of chlipid nanocomplexes (CNs)

Water-soluble chitosan (1 mg/ml) was mixed with Lipofectamine 2000 (Invitrogen) in the ratio of 2:1 (2 μg of chitosan to 1 μg of Lipofectamine in a total volume of 20 μl) and vortexed for 15 min at room temperature. Later these chlipid nanocomplexes were mixed with the respective DNA (1 μg each or total psiRNAs) for *in vitro* studies. The mixtures containing CNs with DNA or psiRNAs were vortexed for 30 min at room temperature before administration.

### Construction of vector-driven siRNAs to inhibit HIV89.6 infection

Six different siRNAs targeting HIV-1 *tat*, *rev*, *gag* and 5’-LTR and rhesus macaque CCR5 and CXCR4 were designed and cloned into the pSilencer-1.0 vector (Ambion), according to the manufacturer’s specifications. These siRNAs were developed based on recombinant simian-human immunodeficiency virus, SHIV89.6p chimeric lentiviruses: namely, SHIVs consisting of a SIVmac239 backbone and HIV type 1 (HIV-1) envelope (env) and regulatory genes. HIV-specific siRNAs were cloned between the *Bam*HI and the *Hind*III sites of pSilencer1.0-U6 (Ambion). When the top strand and the bottom strand of each siRNA-encoding DNA oligonucleotides were synthesized, the *Bam*HI site was included in the 5’-end of siRNA with 10 additional random nucleotides as flanking sequences, and the *Hind*III site was included in the 3’-end of siRNA with 10 other nucleotides as flanking sequences. Each siRNA contained a 21-nt siRNA sense element, which is identical to the targeted gene sequences, then a 8-nt loop region, followed by 21 nt of the antisense elements and a double-T overhang. The siRNA targeting sequences (the sense element) are at position of 142 nt of LTR, 59 nt of Gag, 57 nt of Tat and 42 nt of Rev in the HIV89.6 genome, and 70 nt of rhesus macaque CCR5 and 97 nt of rhesus macaque CXCR4. The top strand and the bottom strand of each siRNA-encoding oligonucleotide were annealed and then digested with Apa1 and EcoR1 before cloning into pSilencer1.0-U6. The primer pairs for the respective siRNAs are shown in Table 1.

**Table 1:** List of genes and siRNA primer sequences.

Name of the gene	Gene ID	Species derived from	Primer pairs
CCR5CCR5-70 a & b	*DQ499965*.*1*	Macaca mulatta isolate CCR5-2	**5’TGTGAAACAAATCGCAGCCCTCGAAATGGCTGCGATTTGTTTCACATTGAATTCCCATGG 3’**
			**5’AATGTGAAACAAATCGCAGCCATTTCGAGGGCTGCGATTTGTTTCACAGGGCCCCATATG 3’**
CXCR4-97 a & b	*U73740*.*1*	Macaca mulatta CXCR4 mRNA	**5’TGCTCATTTCAATAGGATCCTCGAAATGATCCTATTGAAATGAGCATTGAATTCCCATGG 3’**
			**5’AATGCTCATTTCAATAGGATCATTTCGAGGATCCTATTGAAATGAGCAGGGCCCCATATG 3’**
LTR-142 a & b	*U89134*.*1*	SHIV-89.6P	**5’AGCCCTCTTCAATAAAGCTCTCGAAATAGCTTTATTGAAGAGGGCTTTGAATTCCCATGG 3’**
			**5’AAAGCCCTCTTCAATAAAGCTATTTCGAGAGCTTTATTGAAGAGGGCTGGGCCCCATATG 3’**
Gag-59 a & b	*U89134*.*1*	SHIV-89.6P	**5’TTAGGCTACGACCCAACGGCTCGAAATCCGTTGGGTCGTAGCCTAATTGAATTCCCATGG 3’**
			**5’AATTAGGCTACGACCCAACGGATTTCGAGCCGTTGGGTCGTAGCCTAAGGGCCCCATATG 3’**
Tat-57 a & b	*U89134*.*1*	SHIV-89.6P	**5’CTGCTTGTACCAATTGCTACTCGAAATTAGCAATTGGTACAAGCAGTTGAATTCCCATGG 3’**
			**5’AACTGCTTGTACCAATTGCTAATTTCGAGTAGCAATTGGTACAAGCAGGGGCCCCATATG 3’**
Rev-42 a & b	*EF672090*.*1*	SHIV-89.6P	**5’CAGTCAGACTCATCAAGCTCTCGAAATAGCTTGATGAGTCTGACTGTTGAATTCCCATGG 3’**
			**5’AACAGTCAGACTCATCAAGCTATTTCGAGAGCTTGATGAGTCTGACTGGGGCCCCATATG 3’**

HEK293 cells were co-transfected with the 1 μg of each siRNA above with 1 μg of HIV89.6 proviral DNA. The viral supernatant was collected 48 h after transfection and the p24 levels were measured with the HIV1 p24 ELISA kit (Beckman Coulter). Similarly, human PBMC cultures grown in 6-well plates were co-transfected by electroporation with 1 μg of each vector-driven siRNA plus 1 μg of HIV89.6 proviral DNA and the viral supernatant collected 48 h after transfection was examined for p24 levels by HIV-1 p24 ELISA.

### Cell studies using CNs

HEK293 cells grown on 96-well plate were co-transfected with 1 μg of each vector-driven siRNA (psiLTR, psiTat, psiRev, psiGag) or pU6 vector control, along with 1 μg of HIV89.6 proviral DNA using CNs. The viral supernatant was collected 48 h after transfection and the p24 levels were measured using HIV1 p24 ELISA kit (Beckman Coulter) and normalized against the negative control.

### 3D VEC model and anti-HIV studies using CNs

Three-dimensional (3D) vaginal ectocervical tissue was procured from Mat-Tek Corporation and the tissues were maintained as per the manufacturer’s instructions. The tissues were transfected with chitosan or Lipofectamine 2000 or CNs combined with pEGFP. At 48 h post-transfection, the tissues were fixed in 10% formalin, paraffin-embedded, and immunostained using anti-GFP antibody or anti-Ki67antibody tagged with Alexa Fluor 555 (Invitrogen) and nuclear-stained with DAPI. The tissues were also co-transfected with either cocktail plasmid siRNAs-CNs or pU6 vector control-CNs or CNs alone without any plasmids along with p89.6 proviral DNA for 24 h, washed with PBS and fresh medium was added. A p24 ELISA was performed on supernatants collected on days 1, 2, 3, 4 and titrated for p24 concentration (pg/ml).

### Safety studies of the CNs in mice and rabbit vaginal irritation (RVI) model

The safety of systemically administered CNs was evaluated in female 8-week-old Balb/c mice (Jackson Laboratory, Bar Harbor, ME; n = 4/group), which were maintained in pathogen-free conditions at the University of South Florida College of Medicine vivarium. Mice were given intranasally (i.n.) 10 μg of Lipofectamine + 10μg of plasmid DNA encoding enhanced green fluorescence protein (EGFP). Mice were sacrificed on day 4 and their lungs were lavaged with 1 ml of PBS introduced through the trachea. The BAL fluid was centrifuged (10 for min at 300 x g) and the cells were rinsed with PBS and re-suspended. Mice were given PBS as control. Pooled cells from four mice were also quantified by flow cytometry to determine the EGFP transfection levels in BAL cells. Mice BAL fluid was analyzed for IL-6 content using an ELISA (R & D Systems, Minneapolis, MN).

An improved RVI model was used to evaluate microbicide formulations for their effects in tissues by analyses of histopathology, cellular infiltration, apoptosis, and mucosal permeability. In cervicovaginal lavages (CVL), supernatants can be evaluated for cytokines and leukocyte markers, and the cell pellet can be used to quantitate, phenotype, and measure the activation status of the leukocytes. In this study, chlipids and plasmid siRNA cocktail mixed with an anti-inflammatory cream (C5-Biotechnology Inc., Tampa, FL) were administered intravaginally to healthy rabbits. The increase in the number of CD45+ leukocytes in the CVL was determined at the termination of treatment. Three groups consisting of two female rabbits each were maintained under the same environmental conditions and dosed daily with either 10 mg of cream alone or cream plus CNs or a mixture of cream plus CNs containing plasmid siRNA cocktail (consisting of siCCR5, siCXCR4, siTat, siRev, si5’LTR and siGag; 4 μg per plasmid /animal) for 10 days. Serum (3 ml) and CVL fluid (5 ml) were collected from all the rabbits on days 0, 3, 7, 11, and 14 after treatment.

### Safety of systemically administered CNs in nonhuman primates

The safety of systemically administered CNs was assessed in a nonhuman primate, rhesus monkeys via i.p. and oral routes. Eight female rhesus monkeys (5–9 kg) were divided into two groups. Blood was drawn from each animal on day 0 for baseline values of parameters for toxicity studies. Animals in the treatment group (n = 4) were injected i.p. with 1 ml (35 mg/ml) chitosan solution once and then fed with 35 mg of chitosan daily for 2 weeks. Control group (n = 4) animals were injected i.p. with 1 ml of PBS with no feeding of chitosan. Additional blood was drawn on days 16, 36 and 66 after chitosan feeding and blood chemistry was determined for individual animals.

### Determine the Efficacy of psiRNA cocktail combined with CNs in rhesus monkeys infected with SHIV SF162

To assess efficacy, a plasmid siRNA cocktail consisting of representative siCCR5, siCXCR4, siTat, siGag, si5’LTR and siRev (25 μg per plasmid /animal) was evaluated for its efficacy to prevent HIV infection in rhesus monkeys. A group of five cycling rhesus monkeys weighing 6–9 kg and negative for type D retrovirus, SIV and simian T-lymphotropic virus (STLV) were selected for the *in vivo* experiment. All macaques used were pretreated with a single intramuscular dose of Depo-Provera (30 mg/animal) 30 days before the study. The group of five macaques was divided into 2 control and 3 treatment monkeys. A mixture of cream (20 mg) and CNs containing psiRNA cocktail were applied vaginally on days 1, 3 and 6. The control group received a mixture of cream and CNs. On day 6 all the animals were inoculated vaginally with 250 μl (3000 TCID50/ml) of the SHIV SF162-p3 (procured from NIH). All the animals were bled periodically (days 1, 3, 6, 9, 12, 20, 34 and 42) and plasma viral load was measured. Viral RNA was isolated from 200 μl of the plasma collected into EDTA-containing vials, according to the instructions provided by the manufacturer (HighPure viral RNA kit, Roche). Briefly 10 μl of the isolated RNA was subjected to reverse transcription and PCR amplification of a region of *gag* [26]. Later, 10 μl of the amplified product was quantified by anti-DIG enzyme-linked immunosorbent assay (PCR enzyme-linked immunosorbent assay DIG detection, Roche), according to the manufacturer’s instructions, using the probe at 20 nM concentration: 5’-CAT TTG GAT TAG CAG AAA GCC TGT TGG AGA ACA AAG AAG GATGTCAA-3’. All the samples were tested in duplicate. The standard used was a culture supernatant containing free SHIV SF162 aliquoted and diluted in plasma from a sero negative macaque collected in EDTA. All five macaques (2 control and 3 treatment animals) were infected with SHIV and the titer was determined by RNA copies/ml post infection. Viral titers were measured by the absorbance readings at 405 nm both before and after infection. Viral RNA copy numbers were estimated based on a standard curve.

## Results

### Development of CNs as gene delivery vehicles

**Fig 1:**
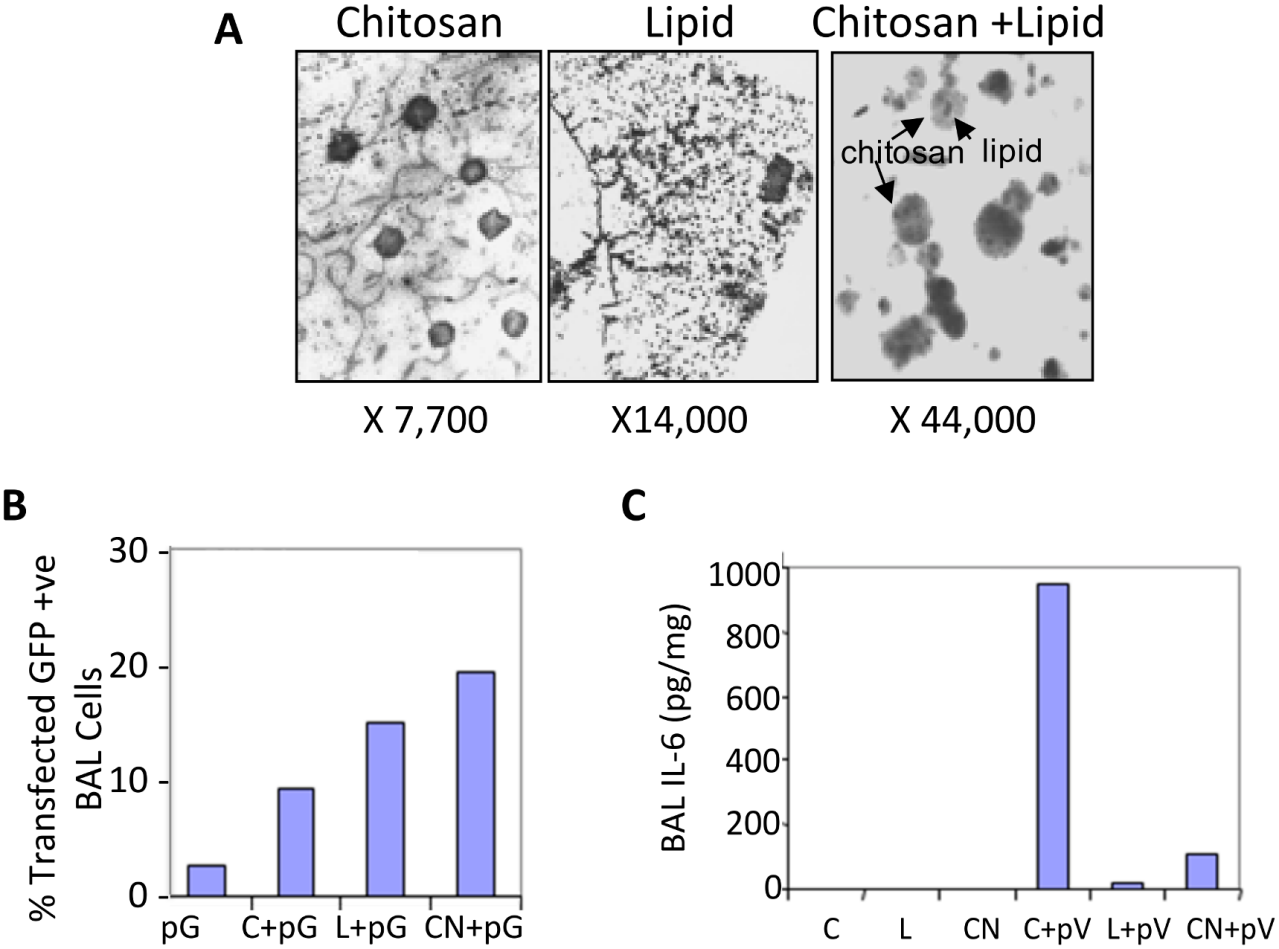
Efficacy and safety of chlipids. (A) Transmission electron micrographs of chitosan, Lipofectamine and CNs complexed with pEGFP. (B) Distribution and quantification of *in vivo* transfection of pEGFP in BAL cells of C57/BL6 mice. pG: Naked EGFP plasmid; C+pG: chitosan-complexed pG; L+pG: Lipofectamine-complexed pG; CN+pG: chlipid-complexed pG. (C) Quantification of IL-6 in BAL following intranasal administration of nanoparticles. C: chitosan; L: Lipofectamine; CN: chlipid; C+pV: chitosan-complexed pVAX DNA; L+pV: Lipofectamine-complexed pVAX DNA; and CN+pV: chlipid-complexed pVAX DNA.

Chitosan (1 mg/ml) and Lipofectamine 2000 (Invitrogen) were mixed at a ratio of 2:1 to form typical CNs and were viewed under an electron microscope. This suggested that CNs had a smaller size (441 nm) compared to the parent chitosan polymer (~1000 nm) (Fig 1A). Further, CNs were evaluated for their transfection efficiency *in vivo* using a mouse lung lavage assay. Plasmid encoding EGFP was complexed with chitosan, Lipofectamine or CNs and administered intranasally and 72 h later BAL cells were collected and GFP expression in the pooled lung lavage were examined by flow cytometry. CNs showed 30% transfection efficiency, as opposed to 20% with classical chitosan or Lipofectamine, and only 10% with naked DNA (Fig 1B). Moreover, very little IL-6 was found in the BAL of CN-complexed pEGFP group compared to chitosan-complexed group (Fig 1C).

### Inhibition of HIV-1 replication using *in vitro* cell culture models

**Fig 2:**
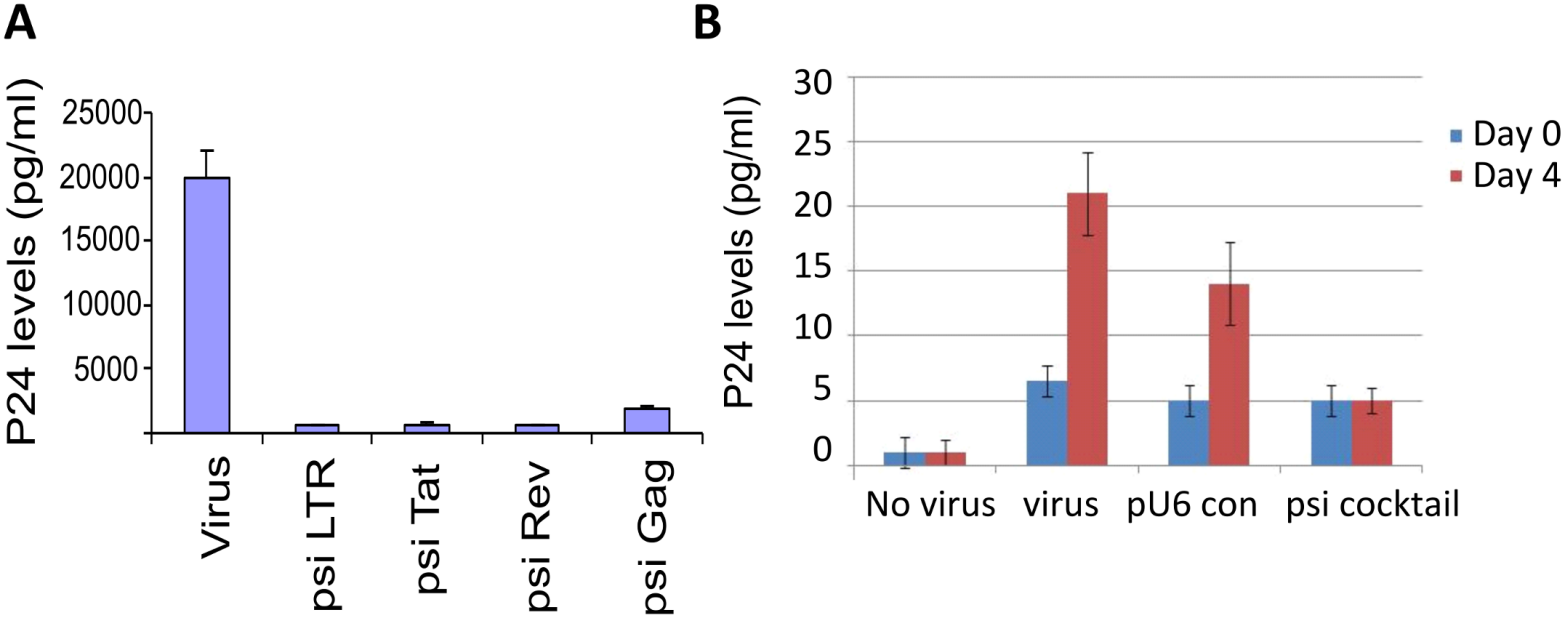
Inhibition of HIV-1 replication by siRNAs targeting HIV-1 genes. HEK293 cells grown in 96-well plates were co-transfected with 1 μg of each vector-driven siRNA (psiLTR, psiTat, psiRev, psiGag) or pU6 vector control, along with 1 μg of HIV89.6 proviral DNA using the Lipofectamine reagent. The viral supernatant was collected 48 h after transfection and the p24 levels were measured by HIV-1 p24 ELISA and normalized against the negative control. The experiment was repeated at least twice and values represent mean ± SEM. (B) Human PBMCs were cultured in 12-well plates, incubated with 1 μg /1 million cells of psicocktail (psiTat + psiRev + psiGag + psiLTR) CNs or pU6 vector control-CNs or naked CNs for 24 h and then infected with HIV89.6 (pg/ml) for 2–3 h. After infection, the cells were washed with PBS and fresh medium was added. Culture supernatants in duplicate were assayed for p24 concentration (pg/ml) by p24 ELISA.

HEK293 cells grown on 96-well plate were co-transfected with 1 μg of each vector-driven siRNA (psiLTR, psiTat, psiRev, psiGag) or pU6 vector control, along with 1 μg of HIV89.6 proviral DNA using Lipofectamine. The viral supernatant was collected 48 h after transfection and the p24 levels were measured by HIV1 p24 ELISA and normalized against the negative control. The screening led to the identification of siRNA plasmids that gave at least a three-log decrease in HIV titer, as measured by p24. The results showed significant reductions in p24 titers with individual psiRNAs (Fig 2A). When psiCXCR4 and psiCCR5 were used independently, there was no significant decrease in viral titers (data not shown).

In contrast to the HEK-293 cell study, human PBMC cultures grown in 12-well plates were incubated with a single dose (1 μg/1million cells) with psiRNA cocktail with CNs (psicocktail) or pU6 vector control-CNs (pU6.con) or CNs alone without any plasmids (Virus) and then infected with HIV-89.6 (final concentration of 15 pg/ml) for 2–3 h. The virus was removed 2 h after the infection by centrifugation, the cells washed and fresh medium was added. Supernatants were collected on days 0, and 4 and p24 levels were determined by HIV-1 p24 ELISA. The results suggest that cocktail psiRNAs decreased HIV titers significantly by day 4 compared with the other panels (Fig 2B).

### Evaluation of psiRNAs using 3D vaginal ectocervical epithelial cell model (3D-VLC-FT model)

**Fig 3:**
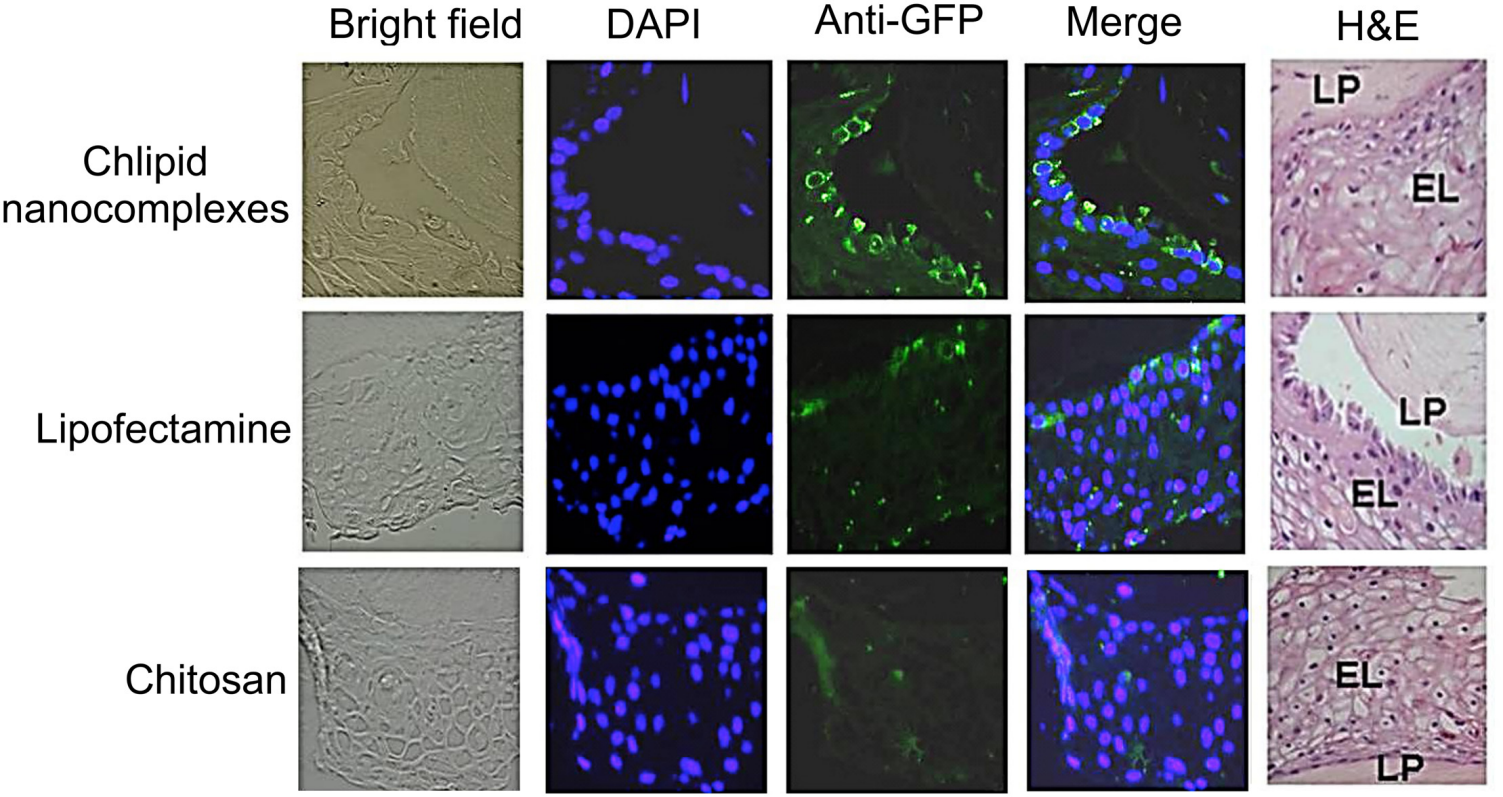
Transfection of cells in a 3D-VEC model. Cells in the 3D-VEC model were transfected with either chitosan or Lipofectamine 2000 or CNs complexed with pEGFP. At 48 h post-transfection, the tissues were fixed in 10% formalin, paraffin-embedded, and immunostained using anti-GFP antibody and nuclear-stained with DAPI. H&E stained histological (formalin-fixed) cross-sections of VLC-FT are also shown. LP, lamina propria; EL, epithelial layers.

3D VLC-FT provides an ideal surrogate model for testing CNs microbicides for targetability, safety, and efficacy. We tested the potential of CNs for delivering pEGFP to various cells in 3D VLC-FT model established using normal human ectocervical cells (NHEC). Cultures were maintained in the air-liquid interface. The tissue consisted of epithelial cells (EL), DCs, fibroblasts and lamina propria (LP). Cultured 3D VLC-FT tissue sections were incubated with three different complexes: chitosan, lipid or CNs complexed with pEGFP. At 48 h later, tissues were fixed, paraffin-embedded and immunostained for anti-GFP antibody. In this experiment, we showed that CNs deliver the marker plasmid more efficiently compared than chitosan or lipids complexed with pEGFP (Fig 3).

**Fig 4:**
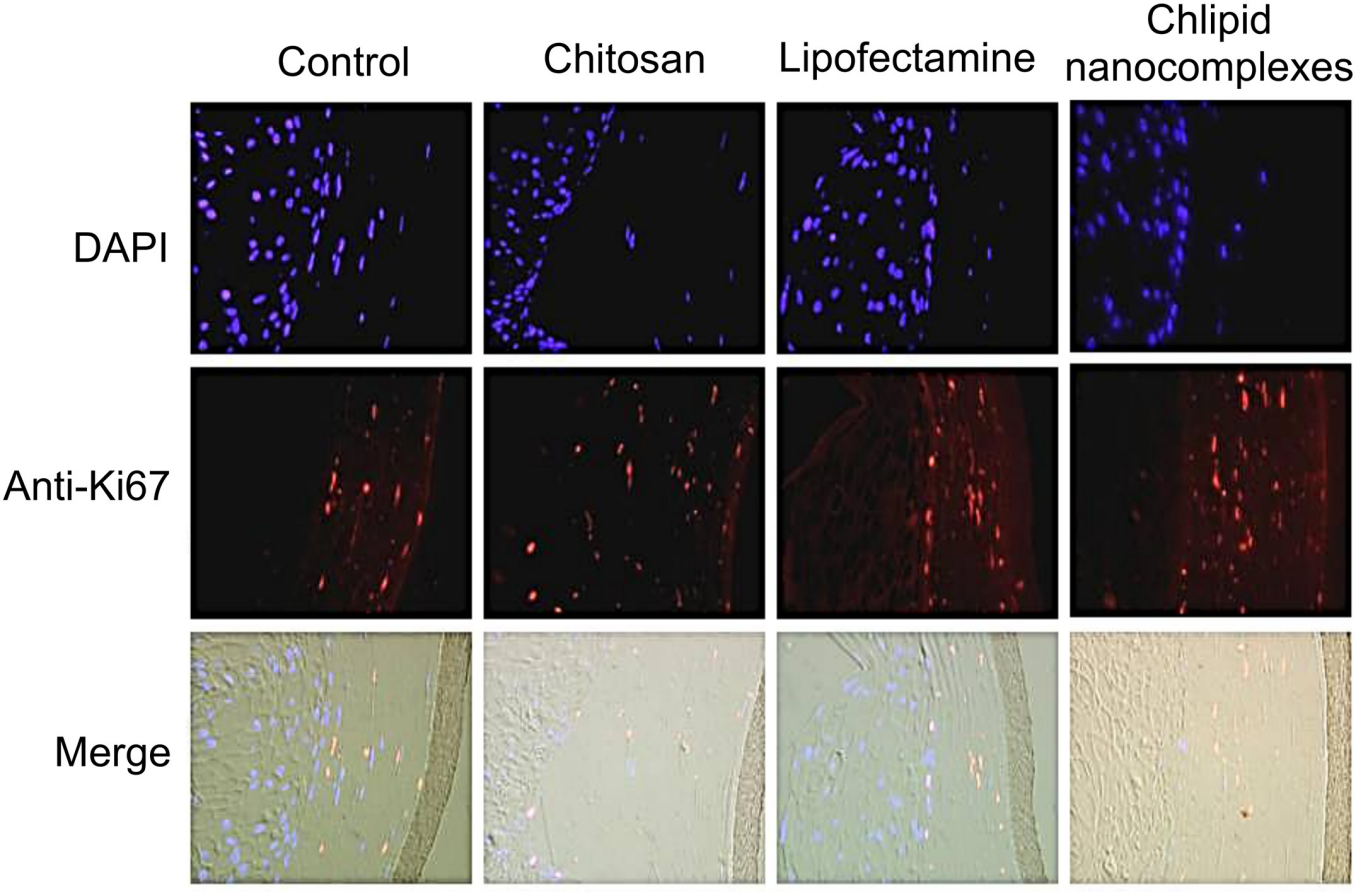
Effects of CNs on the cell proliferation of the 3-D VEC model. Cells in the 3D-VEC model were transfected with either chitosan or Lipofectamine 2000 or chlipid nanocomplexes. At 48 h post-administration, the tissues were fixed in 10% formalin and paraffin embedded. Sections were immunostained using anti-Ki67antibody tagged with Alexa Fluor 555 and DAPI stained.

To determine whether transfection of CNs in 3D-VEC culture altered cell proliferation, the tissue sections were incubated with chitosan, lipid or CNs. At 48 h later, tissues were fixed, paraffin embedded and immunostained for anti-Ki67 antibody tagged with Alexa Fluor 555. Results showed that the use of CNs as a transfection reagent had no effect on cell proliferation as shown by Ki67 staining compared with the control or the cells treated with chitosan or lipid (Fig 4).

**Fig 5:**
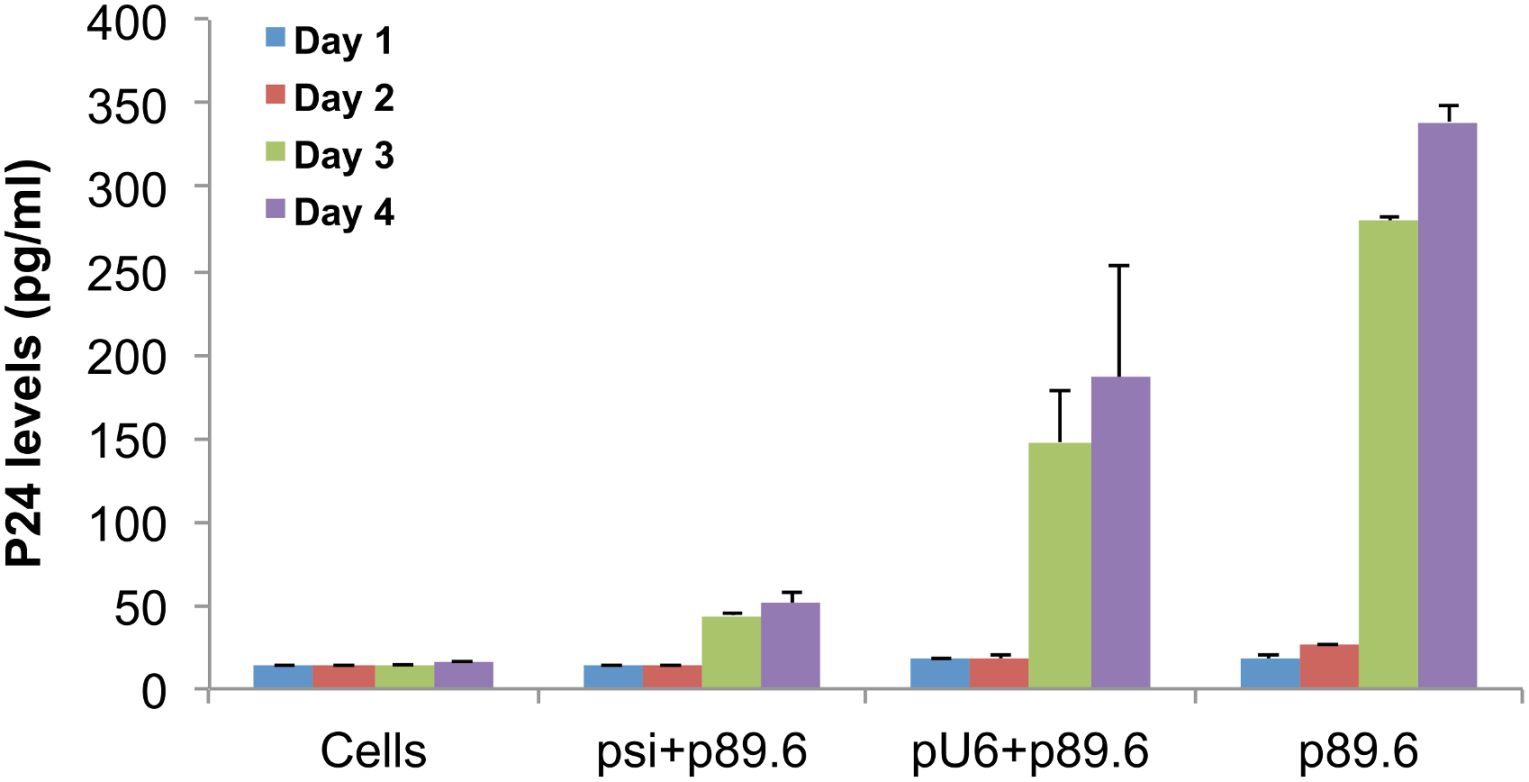
Inhibition of HIV-1 replication by siRNAs using 3D VEC model. The 3-D VEC tissues were co-transfected with cocktail plasmid siRNAs-chlipid nanocomplexes or pU6 vector control-chlipid nanocomplexes or chlipid complexes alone with no plasmids. A p24 ELISA was performed on supernatants collected on days 0, 2, and 3 post-infection and p24 concentrations (pg/ml) were measured using p24-ELISA. Assays were run in duplicate on supernatants collected and expressed as mean p24 concentration in pg/ml ± SEM.

The 3-D VEC tissues were incubated with either cocktail plasmid siRNAs-CNs (psi cocktail) or pU6 vector control-CNs (pU6 con) or CNs alone without any plasmids (Virus) for 24 h and then infected with HIV-89.6 (final concentration of 15 pg/ml) for 2–3 h, washed with PBS and fresh medium was added. The no virus control is the normal tissue control. Supernatants were collected on days 0, 2, 3 and titrated for p24 concentration (pg/ml) using a p24-ELISA. From the virus titer measured from supernatants collected on days 2 and 3, treatment with psicocktail showed significant reductions in virus titer when compared with the pU6 con or CNs alone. The results suggested that administration of the cocktail of plasmid siRNAs complexed with CNs significantly reduced the virus titer (as measured by p24 ELSIA in pg/ml) by day 3, while in the pU6 control, the virus titer had plateaued at days 2, and 3 (Fig 5).

### Safety studies in RVI model

**Fig 6:**
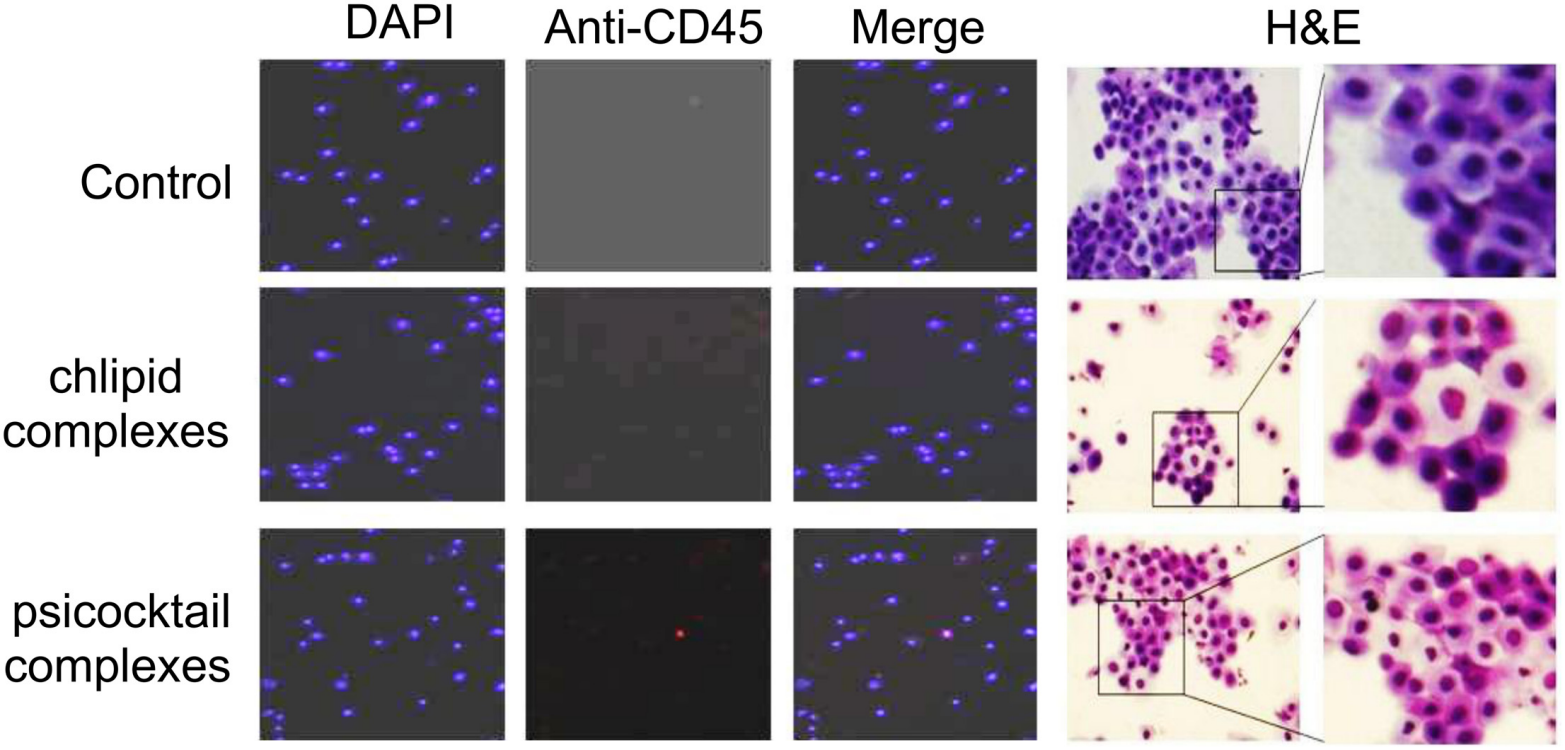
Safety studies in RVI model. Rabbits (n = 9) were divided into three groups and in each group (n = 3) we applied cream alone, cream mixed with chlipid nanocomplexes, or cream mixed with psicocktail chlipid nanocomplexes every day for 9 days. In the RVI model, the CVL was collected at days 11 and 20, the cells were cytospun, fixed with 4% paraformaldehyde and permeabilized using 100% methanol. The cells were later treated with anti-CD45 tagged with Alexa Fluor 555 and viewed under a fluorescence microscope. Images were taken at 100x magnification. The two panels on the right show H&E-staining of the cells from CVL of rabbits treated as described above.

Three groups consisting of two female rabbits each were maintained under the same environmental conditions and dosed daily with either 10 mg of cream alone or cream plus CNs or a mixture of cream plus chlipids containing plasmid siRNA cocktail (consisting of siCCR5, siCXCR4, siTat, siRev and siGag; 4 μg per plasmid /animal) for 10 days. Serum (3 ml) and CVL (5 ml) fluid were collected from all rabbits on days 0, 3, 7, 11, and 14 after the treatment. None of the cells from rabbit lavages treated with cream alone or cream plus CNs or a mixture of cream plus chlipids containing plasmid siRNA cocktail showed CD45-positive staining, indicating that the rabbits did not have any inflammation or irritation. H&E staining of the cells from rabbit CVL, 11 days after treatment with a cocktail of plasmid siRNAs complexed with chlipids formulated as cream showed no neutrophil infiltration (Fig 6).

### Efficacy of cream and plasmid siRNA cocktail complexed with CNs in rhesus monkeys infected with SHIV SF162

Before evaluating the effects of the plasmid siRNA cocktail, we evaluated the systemic safety of the chitosan nanoparticles in a nonhuman primate by administering chitosan to rhesus monkeys via i.p. and oral routes. Table 2 summarizes the blood chemistry data averaged for each group. The results suggested that treatment with chitosan nanoparticles did not cause toxicity in rhesus monkeys with respect to proteins (total protein, albumin, bilirubin, alkaline phosphatase, aspartate amino-transferase, amylase, gammaglutamyl transpeptidase), glucose, creatinine, lipids (triglycerides and cholesterol) and inorganic elements (urea nitrogen, calcium, phosphorous, sodium, chloride and magnesium).

**Table 2:** Blood chemistry of control and nanocomplexes treated monkeys.

Control Group					Nanocomplex Treated Group			
Date of blood drawn	0	16	36	66	0	16	36	66
**Glucose**	84.5	79.3	80.3	84.0	80.75	79.75	75.5	64.5
**Urea Nitrogen**	18.8	16.3	16.3	24.0	14.5	16	15.5	17
**Creatinine**	0.8	0.7	1.0	1.0	0.775	0.625	0.925	1.025
**Total Protein**	7.8	7.5	7.6	7.1	8.05	7.575	7.55	7.575
**Albumin**	4.6	4.5	4.7	4.4	4.325	3.95	4.25	4.475
**Total Bilirubin**	0.1	0.2	0.2	0.2	0.1	0.125	0.125	0.1
**Alkaline Phosphatase**	73.0	92.5	93.3	85.5	78.5	130.25	105.75	109.5
**AST (SGOT)**	32.3	32.8	31.3	55.8	25	42.25	33.5	28.25
**Cholesterol**	140.5	139.3	139.0	139.8	178.25	175.25	178.25	182.25
**Calcium**	10.0	9.9	9.8	9.4	10.25	9.9	10.025	9.85
**Phosphorous**	3.5	3.5	3.3	4.0	3.65	4.125	4.275	3.675
**Sodium**	149.8	147.5	151.8	154.3	146.25	147.75	151.75	155.25
**Potassium**	9.7	3.8	3.5	3.7	16.65	3.925	3.85	3.925
**Chloride**	108.3	108.5	110.0	110.3	103.5	107	109	110.25
**Albumin/Globulin Ratio**	1.4	1.5	1.6	1.7	1.175	1.1	1.325	1.475
**BUN/Creatinine Ratio**	24.0	24.8	16.5	25.5	19.25	25.75	16.75	16.75
**Globulin**	3.3	3.0	2.9	2.7	3.725	3.625	3.3	3.1
**Amylase**	275.5	308.3	303.0	312.8	254	328.25	313.75	330.75
**Triglycerides**	70.5	115.5	81.5	74.5	82.5	85	78.5	94.25
**GGTP**	45.3	42.8	46.5	46.3	31.75	30	33.5	38
**Magnesium**	1.6	1.7	1.7	1.8	1.65	1.65	1.775	1.825

**Fig 7:**
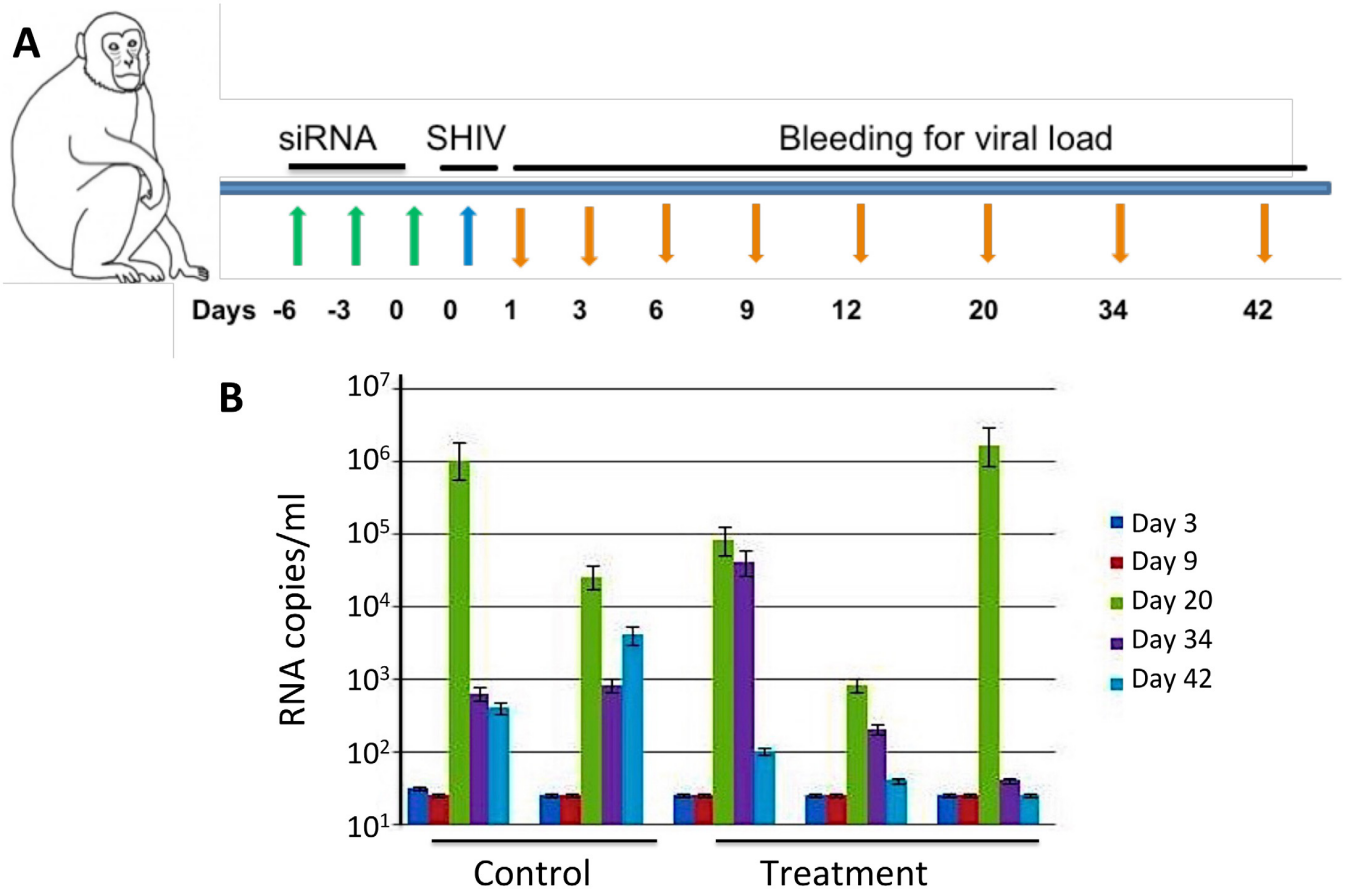
Efficacy of psi cocktail CNs in rhesus monkeys infected with SHIV SF162. (A) Schematic of the protocol used. (B) Treatment of monkeys with a mixture of cream and chlipid nanocomplexes containing plasmid siRNA cocktail resulted in the reduction of viral titers in SHIV SF162 -infected monkeys. Viral RNA was isolated from 200 μl of the plasma (HighPure viral RNA kit, Roche). Isolated RNA was subjected to reverse transcription and PCR amplification of a region of *gag*, and the amplified product was quantified by anti-DIG enzyme-linked immunosorbent assay. All the samples were run in duplicate.

The efficacy of the CNs complexed with plasmid siRNA cocktail consisting of representative siCCR5, siCXCR4, siTat, siGag, si5’LTR, siRev (25 μg per plasmid /animal) to prevent SHIV infection was examined in a pilot study using rhesus monkeys (Fig 7A). A cream formulation of CNs containing psiRNA cocktail was applied vaginally on days -6, -3 and 0. On day 0 all the animals were inoculated intravaginally with SHIV SF162-p3 viral stock. All the animals were bled periodically on days 1, 3, 6, 9, 12, 20, 34 and 42 post virus challenges. Plasma viral load was measured by PCR enzyme-linked immunosorbent assay. Results suggested that administration of the cocktail of plasmid siRNAs complexed with CNs prior to infection significantly reduced the viral load of the treated monkeys by day 42 of infection, while in the control monkeys, the viral load had plateaued following days 34 and 42 (Fig 7B).

## Discussion

A review of preclinical and clinical research on the development of microbicides formulated to prevent vaginal HIV transmission yielded 118 studies; 73 preclinical and 45 clinical [27]. Currently, microbicide development efforts are focusing on compounds with highly potent and HIV-specific mechanisms of action, combination products, novel formulations and carefully designed pharmacokinetic and pharmacodynamic evaluations [28]. All of the products in phase III are non-specific microbicides, which have been shown to be moderately effective in preliminary or reported studies. The latest Phase III study of PRO 2000 in 9,400 women showed that PRO 2000 does not offer protection against HIV. However, a study that enrolled 889 women in South Africa suggests that a vaginal microbicide containing tenofovir may be effective [29]. The sample size is relatively small, which restrict the finding that tenofovir reduces the risk of HIV transmission in heterosexual women. Moreover, microbicides based on the HIV-1 neutralizing antibody VRC01 or the combination of four first-generation broad neutralizing antibodies (bNAbs)-b12, 2F5, 4E10, and 2G12, showed protection against HIV-1 challenge in humanized BLT mouse model [30]. These studies suggested that we should not continue our investigation focused on the early generation, novel non-specific microbicides. These results also raise little doubt that at this juncture, agents with specific action against HIV, such as anti-retroviral drugs or targeted siRNAs, offer the most hope.

RNAi is a well-characterized phenomenon that has proven effective in silencing a number of genes of different viruses [31]. siRNAs can be introduced into cells as synthetic doubled-stranded oligonucleotides. Previously, others have developed DNA vector-based approaches to introduce siRNA into tissue culture systems and *in vivo* against HIV, HCV, RSV, SARS, and dengue virus as well as other viruses [32–46]. siRNAs are used to inhibit HIV-1 by targeting various viral proteins or cellular cofactors that are important for HIV replication. The cellular receptor CD4, co-receptor CCR5, and viral Tat, Rev and Gag proteins are among the successful targets first reported in 2002 [34, 35, 37, 39, 41]. Other siRNAs targeting CXCR4, DC-SIGN, human transcription elongation factor P-TEFb or cyclophilin A were also shown to be effective in inhibiting HIV-1 replication *in vitro* [32, 33, 36, 38, 40, 43, 44, 46]. siRNAs can be introduced into cells as synthetic doubled-stranded RNAs, but are more effective when expressed from a plasmid under control of the RNA polymerase III promoter. Silencing CD4 reduced CD4 expression in 75% of Magi-CCR5 cells and reduced HIV-1 replication four-fold compared with the control [41]. The degree of inhibition was similar when siRNA-targeting CCR5 was tested in PBLs from different donors (3–7 fold of HIV-1 inhibition) [43]. Some synthetic siRNAs were effective against HIV-1 replication in PBMCs, although the protection was transient. However, when siRNAs targeting CCR5 and *tat* were expressed through lentiviral vectors, primary macrophages could be protected against HIV-1 infection in culture for longer periods [38].

siRNAs targeting of viral proteins, especially the regulatory proteins, Tat and Rev, would be good candidates for gene silencing *in vivo*, except that frequent mutations in the HIV-1 genome could abolish their effectiveness. Thus, a cocktail of siRNAs targeting different HIV-1 gene sequences would be more likely to avoid HIV-1 mutant escape. By including siRNAs targeting HIV-1 coreceptors of CCR5 or CXCR4, additional advantages can be achieved to prevent HIV-1 transmission, because siRNA targeting HIV-1 coreceptors are not affected by mutations of the HIV-1 viral genome. Silencing of DC-SIGN by siRNA also significantly inhibited HIV-1 replication, presumably by preventing the transfer of virus from DCs to T cells. DC-SIGN is an additional target in controlling HIV-1 for our future optimization of psiRNA cocktail [32]. Although the viral titer was significantly lower in the group treated by siRNA-based microbicide around day 42, complete block of SHIV infection was obtained in our studies. The efficacy of our nanomicrobicides may have been restricted by the SHIV/macaque model, because siGag and siLTR are both target HIV sequences instead of SIV sequences. An alternative option is to test the efficacy of our nanomicrobicides in humanized BLT mice challenged by HIV-1, as humanized mouse model have been recognized as an easy approach with much potential for vaccine studies [47–54].

Preclinical safety and efficacy testing of microbicide candidates involves a large number of *in vitro*, *ex vivo*, and *in vivo* assays and models. Here we demonstrated that chitosan-based nanoparticles showed no significant toxicity in rhesus monkeys, which is consistent with other modified chitosan nanoparticles for siRNA delivery [55, 56]. Thus, the siRNA-based chlipid microbicides described in this study were subjected to a very cautious and rigorous preclinical evaluation to assess their efficacy in models used for microbicide development.
